# Ets family proteins regulate the EMT transcription factors Snail and ZEB in cancer cells

**DOI:** 10.1002/2211-5463.13415

**Published:** 2022-04-29

**Authors:** Mai Koizumi Ichikawa, Kaori Endo, Yuka Itoh, Asami Hotta Osada, Yujiro Kimura, Koichiro Ueki, Kunio Yoshizawa, Keiji Miyazawa, Masao Saitoh

**Affiliations:** ^1^ Department of Biochemistry Graduate School of Medicine University of Yamanashi Chuo‐city Japan; ^2^ Center for Medical Education and Sciences Graduate School of Medicine University of Yamanashi Chuo‐city Japan; ^3^ Department of Oral and Maxillofacial Surgery Graduate School of Medicine University of Yamanashi Chuo‐city Japan

**Keywords:** cancer, EMT, Ets1, snail, TGF‐β, ZEB1

## Abstract

The epithelial–mesenchymal transition (EMT) is a crucial morphological event that occurs during epithelial tumor progression. Snail and ZEB1/2 (ZEB1 and ZEB2), known as EMT transcription factors, are key regulators of this transition. ZEB1/2 are positively correlated with EMT phenotypes and the aggressiveness of cancers. On the contrary, Snail is also correlated with the aggressiveness of cancers, but is not correlated with the expression of EMT marker proteins. Snail is induced by transforming growth factor‐β (TGF‐β), a well‐known inducer of EMT, in various cancer cells. Interestingly, Snail induction by TGF‐β is markedly enhanced by active Ras signals. Thus, cancer cells harboring an active Ras mutation exhibit a drastic induction of Snail by TGF‐β alone. Here, we found that members of the E26 transformation‐specific (Ets) transcription factor family, Ets1 and Ets2, contribute to the upregulation of both Snail and ZEB1/2. Snail induction by TGF‐β and active Ras is dramatically inhibited using siRNAs against both Ets1 and Ets2 together, but not on their own; in addition, siRNAs against both Ets1 and Ets2 also downregulate the constitutive expression of Snail and ZEB1/2 in cancer cells. Examination of several alternatively spliced variants of Ets1 revealed that p54‐Ets1, which includes exon VII, but not p42‐Ets1, which excludes exon VII, regulates the expression of the EMT transcription factors, suggesting that Ets1 is a crucial molecule for regulating Snail and ZEB1/2, and thus cancer progression is mediated through post‐translational modification of the exon VII domain.

## Graphical abstract

Among key EMT transcription factors, ZEB1/2 are positively correlated with the mesenchymal marker proteins, whereas Snail is not correlated with EMT marker proteins, but largely regulates cellular senescence. Importantly, members of the Ets transcription factor family, Ets1 and Ets2, contribute to the upregulation of both Snail and ZEB1/2 in cancer cells.

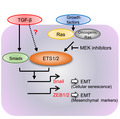

**Abbreviations**
EMT: epithelial–mesenchymal transition
Ets: E26 transformation‐specific
OSCC: oral squamous cell carcinoma
qPCR: quantitative real‐time PCR
SA‐βgal: senescence‐associated β‐galactosidase
siRNA: short interfering RNA
TGF‐β: transforming growth factor‐β
ZEB1: zinc‐finger E‐box binding homeobox

The process of cancer cell invasion and metastasis requires the loss of cell–cell interactions combined with the acquisition of motility, and occasionally, epithelial–mesenchymal transition (EMT) [1]. The EMT process is induced by some transcription factors known as EMT transcription factors (EMT‐TFs), including the Snai1 family (Snail, Slug, and Smuc), the ZEB family (ZEB1 [zinc‐finger E‐box binding homeobox 1] and ZEB2), and basic helix–loop–helix factors (E12/E47 and Twist) [2, 3]. When these factors are aberrantly expressed in cancer cells, they induce EMT and promote the development of metastatic properties such as migration and invasion. Snail and ZEB1/2 (ZEB1 and ZEB2) repress E‐cadherin expression through direct binding to the E‐cadherin promoter region [4, 5]. ZEB1/2 are highly expressed in aggressive basal‐like breast cancers associated with a poor prognosis, while being hardly expressed in the luminal‐type breast cancers associated with a good prognosis [6, 7]. In oral squamous cell carcinoma (OSCC), mesenchymal‐like cells, similar to basal‐like breast cancer cells, express low levels of E‐cadherin and high levels of vimentin and ZEB1/2, while other epithelial‐like cells, similar to luminal‐type breast cancer cells, expressed low levels of vimentin and ZEB1/2 and high levels of E‐cadherin [8, 9].

The E26 transformation‐specific (Ets) family of transcription factors has twenty‐eight members in the human genome and regulates many different biological processes, including cell differentiation, cell proliferation, cellular senescence, angiogenesis, and neoplasia. Among the Ets family of transcription factors, Ets1, a prototypic member of this family, regulates EMT during embryo development of chicken, whereas Ets2 functions redundantly with Ets1 to regulate various cellular phenomena [10, 11]. Ets transcription factors have been identified as mediators of RAS/ERK signaling, and phosphorylation of Ets proteins by ERK can activate transcription. We have also reported that, in murine mammary gland epithelial NMuMG cells, Ets1 enhances ZEB1 promoter activity during EMT induced by transforming growth factor‐β (TGF‐β), a well‐known inducer of EMT during development, fibrosis, and cancer progression [12]. Ets1 activates the *ZEB1* promoter and induces endogenous ZEB expression in breast cancer cells. Silencing Ets1 represses the expression of ZEB1/2 and partially restores sensitivity to anti‐tumor drugs and their epithelial phenotypes [13]. In addition, ZEB expression induced by Ets1 is inhibited by the epithelium‐specific Ets (ESE) transcription factors, ELF3 (ESE1) and EHF (ESE3) [8].

In contrast to ZEB1/2, the expression level of Snail is not closely linked to mesenchymal phenotypes in OSCC and breast cancer cells [7, 8], but Snail is reported to be aberrantly expressed in some types of cancers, and regulates various biological processes such as cell motility, proliferation, cellular senescence, and apoptosis [14]. Snail is upregulated by the TGF‐β–Smad signaling pathway, which is remarkably enhanced through cooperative pathways, such as active Ras signaling [15, 16]. By contrast, Ras signaling unaffected or only slightly inhibited the direct target genes of TGF‐β [17]. Ras and TGF‐β, therefore, cooperate to selectively induce Snail.

We have previously reported that Snail is dramatically induced by TGF‐β in cooperation with active Ras, such as H‐Ras G12V and K‐Ras G12D, and by TGF‐β alone in cancer cells harboring an active K‐Ras mutation. In this study, we hypothesized that Ets1 contributes to the upregulation of Snail and ZEB1/2 in cancer cells. We found that siRNAs against both Ets1/2 (Ets1 and Ets2), but not either alone, repressed expression of Snail induced by TGF‐β in cancer cells with an active K‐Ras mutation. In addition, both siRNAs repress the expression of Snail and ZEB1/2 in cancer cells which have high levels of both Snail and ZEB1/2. Importantly, MEK‐ERK inhibition downregulated Snail, whereas Ets1 with a mutation at ERK‐mediated phosphorylation sites still induced Snail expression. Interestingly, among several alternative splicing variants of Ets1, p54‐Ets1 including exon VII, but not p42‐Ets1 excluding exon VII, activated Snail promoter activity. These findings suggest that Ets1 regulates the expression of both Snail and ZEB1/2 through potential post‐translational modification of exon VII, likely dependent on the MEK‐ERK pathway. Moreover, Snail, rather than ZEB1/2, in OSCC cells suppressed cellular senescence. Therefore, Ets family proteins define the EMT state through the regulation of the EMT transcription factors.

## Materials and methods

### Cell culture and reagents

All cells have been described previously [8]. The authenticated cells by single tandem repeat analysis were cultured in Dulbecco's modified Eagle's medium (DMEM; Nacalai tesque, Kyoto, Japan) containing 500 μg·mL^−1^ streptomycin, 500 units·mL^−1^ penicillin, and 10% fetal bovine serum (FBS) in a humidified atmosphere containing 5% CO_2_ at 37 °C. IMR90 cells were maintaining in Eagle’s minimum essential medium with Eagle’s salts (EMEM; Wako Pure Chemical Industries, Osaka, Japan) containing 1 mm nonessential amino acids (Life Technologies, Carlsbad, CA, USA), 10% FBS, and the same antibiotics. Cell culture supernatants were tested for mycoplasma contamination using the TaKaRa PCR Mycoplasma Detection Set (Takara‐Bio, Kusatsu, Japan). Recombinant human TGF‐β1 was obtained from R&D Systems (Minneapolis, MN, USA). U0126 (#211–01051) and PD98059 (P215) were purchased from Wako and Sigma‐Aldrich (St. Louis, MO, USA), respectively. The following antibodies were obtained from their respective manufacturers: Rabbit monoclonal anti–phospho‐ERK1/2 (#9101S Lot30) and rat monoclonal anti‐Snail (SN9H2 Lot2) from Cell Signaling Technology (Beverly, MA, USA); Rabbit polyclonal anti‐ZEB1 (NBP1‐05987 LotA3) and anti‐ZEB2 (NBP1‐82991 LotB96837) from Novus Biologicals (Littleton, CO, USA); Rabbit polyclonal anti‐Ets1 (sc‐350, Lot#12214) and mouse monoclonal anti‐Ets2 (sc‐365666, Lot#K1717) from Santa Cruz Biotechnology (Dallas, TX, USA); Rabbit polyclonal anti–phospho‐T38 (ab59179, Lot GR84256‐3) from Abcam (Cambridge, UK); Mouse monoclonal anti–α‐tubulin (T9026) from BD Biosciences (Lexington, KY, USA); Mouse monoclonal anti‐Flag (5A8E5) from Nacalai tesque; Rat anti‐HA (3F10) from Roche (Indianapolis, IN, USA).

### Immunoblotting

The procedures used for the immunoblotting assay were previously described [18]. Cells were lysed in a buffer containing 150 mm NaCl, 20 mm Tris/HCl [pH 7.5], 1% Nonidet P‐40, 1 mm EGTA, 5 mm EDTA, phosphatase inhibitors and protease inhibitor cocktails. Protein concentration was measured using BCA protein assay reagent (Thermo Fisher Scientific, Waltham, MA, USA). The collected proteins were separated by SDS/PAGE and transferred to polyvinylidene difluoride membranes (Pall, Glen, Cove, NY, USA). The blots were incubated at room temperature for 1 h. The working dilution of primary and HRP‐conjugated secondary (Jackson ImmunoResearch Laboratories, West Grove, PA, USA) antibodies was 1 : 1000 and 1 : 10 000, respectively. Proteins were visualized using Amersham Biosciences ECL Western blotting detection reagent (GE Healthcare, Chicago, IL, USA). All data were obtained from the image files acquired using a LAS‐4000 mini luminoimage analyzer (Fujifilm, Tokyo, Japan).

### RNA extraction and reverse transcription

Total RNA extracted using the RNeasy mini kit with DNase treatment (Qiagen, Venlo, The Netherlands) was stored at −80 °C until use. After the purity of RNA samples was spectrophotometrically assessed, cDNA was immediately generated by 2 μg of total RNA using the PrimeScript First Strand cDNA synthesis kit (Takara‐Bio).

### Quantitative real‐time PCR (qPCR)

The PrimeScript First Strand cDNA synthesis kit (TaKaRa‐Bio) was used for synthesizing cDNAs from total RNA extracted using the RNeasy mini kit (Qiagen). We performed qPCR analyses using the Power SYBR Green PCR Master Mix (Applied Biosystems, Foster City, CA, USA) and normalized the relative expression level of each mRNA against that of GAPDH mRNA. According to previous reports [19, 20], a standard curve was ensured by producing a twofold dilution series over five points of the most concentrated cDNA sample. We performed qPCR analysis in triplicate for all PCR primer pairs shown in Table S1 and obtained qPCR detection instruments including 96‐well plates from Applied Biosystems.

### Conventional PCR

To design conventional PCR primers using Primer‐Blast, the target sequence was a GC content (45–55%) with 300–500 bp long and melting temperature (55–65 °C). Conventional PCR was performed with LA Taq polymerase (TaKaRa‐Bio). Reaction conditions were 1 min at 95 °C, 20 s at 98 °C, 1 min at 50 °C, and 2 min at 72 °C for 30 cycles, followed by 10 min at 72 °C. A Printgraph AE‐6932GXES gel detection system (ATTO Corp., Tokyo, Japan) was used for visualizing PCR products, which were separated on 2% agarose gels and stained with ethidium bromide. The GAPDH‐encoding gene was used as an internal control.

### RNA interference

Transfection of siRNAs (Stealth RNAi; Invitrogen, Carlsbad, CA, USA, Ets1 [103402, 103403, 103404], Ets2 [40619, 40620], ZEB1 [HSS110549], ZEB2 [HSS114854], and Snail [21]) was carried out in six‐well tissue culture plates using Lipofectamine RNAiMax (Invitrogen). siRNA final concentration was 10 nm.

### Invasion assay

The cells were seeded in triplicate onto cell culture inserts (8.0 µm pore size; BD Falcon, Franklin Lakes, NJ, USA) coated with type I‐C collagen (Nitta Gelatin, Osaka, Japan). Twenty‐four h later, we removed cells that had not invaded into the lower surfaces of the filters using cotton swabs and fixed cells that had invaded into the lower surfaces with acetone and methanol (1 : 1), followed by staining with Trypan Blue. Invasion was quantified by visually counting the photographed cells in several fields, followed by statistical analysis.

### Senescence‐associated β‐galactosidase staining

Senescence‐associated β‐galactosidase (SA‐β‐gal) staining was performed as described previously [21]. Briefly, after cells were fixed in 2% formaldehyde/0.2% glutaraldehyde, the cells were stained with a staining solution (150 mm NaCl, 5 mm potassium ferricyanide, 5 mm potassium ferrocyanide, 2 mm MgCl_2_, 40 mm citric acid/sodium phosphate, and 1 mg·mL^−1^ X‐gal) at 37 °C for 48 h in Ca9‐22 cells and 3.5 h in IMR90 cells. After SA‐β‐gal‐negative and SA‐β‐gal‐positive cells were photographed at 100× magnification, we counted them in five random independent fields.

### Generation and infection of lentiviruses

Ca9‐22 cells stably expressing Snail were established using a lentiviral expression system. cDNA encoding human Snail with a C‐terminal HA epitope tag was subcloned into lentiviral pCSII‐EF/CMV‐RfA vectors using Gateway Technology (Invitrogen). After the lentiviral vectors together with pCAG‐HIVgp and pCMV‐VSV‐G‐RSV‐Rev vectors [15] were transfected into 293FT cells by Lipofectamine 2000 (Invitrogen), the culture media were collected and used for infection into Ca9‐22 cells.

### Luciferase assay

We seeded HeLa cells in duplicate in 24‐well tissue culture plates 18 h prior to transfection. X‐treme Gene HP DNA transfection reagent (Roche) was used for transiently transfection with human Snail promoter‐Luc, pTK‐Renilla (Promega, San Luis Obispo, CA, USA), and the indicated expression plasmids. After treatment with 1 ng·mL^−1^ of TGF‐β for 12 h, luciferase activity was measured using the Dual Luciferase Reporter Assay (Promega) in a luminometer (SpectraMax L Microplate Reader; Molecular Devices, Sunnyvale, CA, USA). The luciferase activity of sea‐pansy from the co‐transfected pTK‐Renilla was used to normalize the luciferase activity of fireflies.

### DNA constructs and mutagenesis

Plasmids used in this study are described elsewhere [13]. Human Ets1 with a point mutation was constructed by PCR‐based mutagenesis [13]. The primers used are shown in Table S1.

### Statistical analysis

The data are presented as mean ± SD. Statistical analyses were performed using Student’s *t*‐test between any two groups.

## Results

### Ets1 and Ets2 (Ets1/2) mediate TGF‐β‐induced Snail expression

**Fig. 1:**
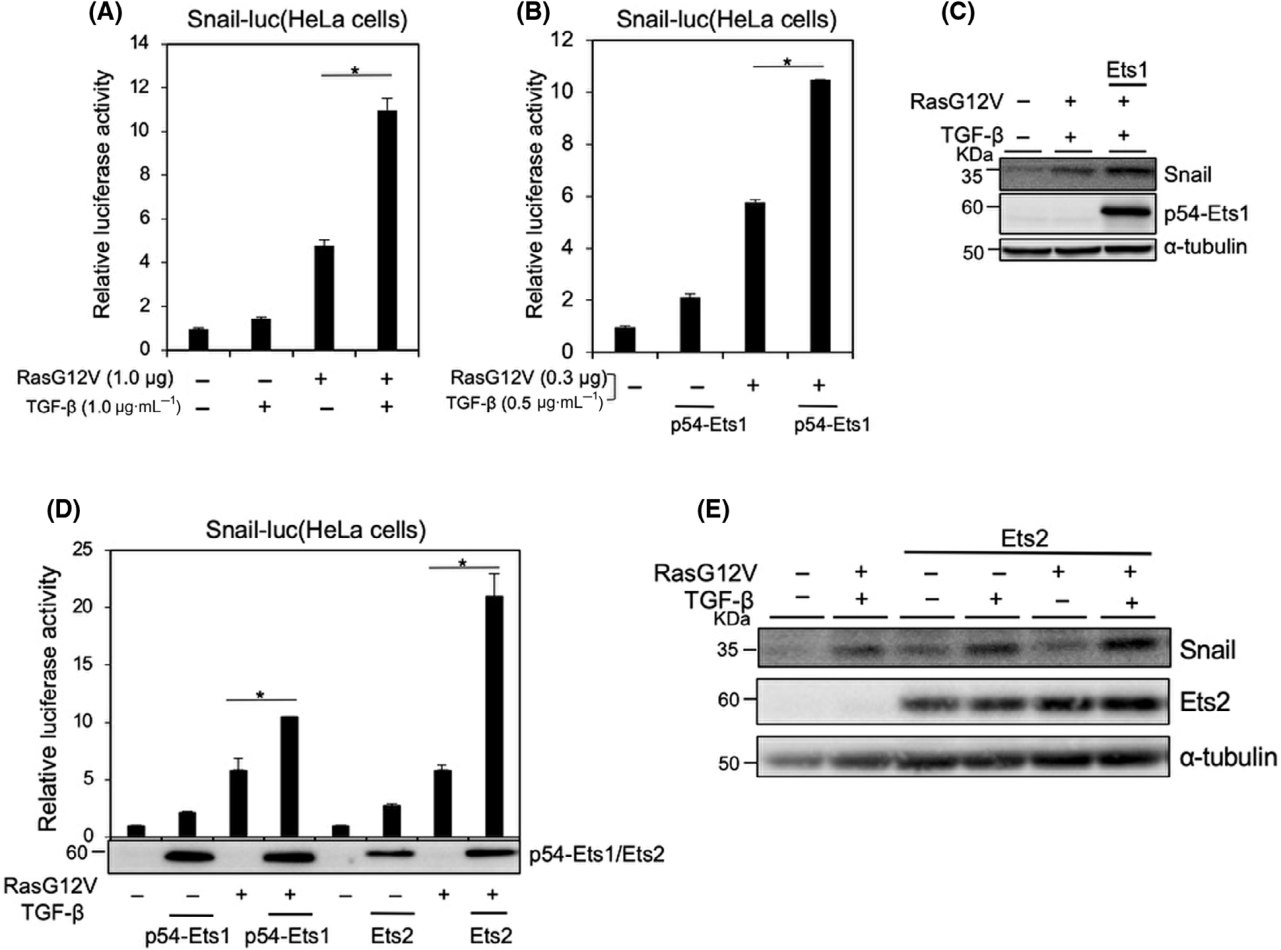
Ets is involved in Snail expression through TGF‐β1 and Ras signaling in HeLa cells. (A, B, D) HeLa cells were co‐transfected with a mouse Snail promoter–reporter construct (Snail‐Luc.) and the indicated expression plasmids. At 8 h after transfection, the cells were treated with TGF‐β1 for an additional 18 h, and the activities of Snail promoters were measured. (C, E) After transfection with the indicated expression plasmids, the cells were treated with 1 ng·mL^−1^ TGF‐β1 for an additional 3 h, followed by immunoblot analysis. Each value represents the mean ± SD of three biological replicates. Similar results were obtained in at least three independent experiments. *P* values were determined by Student’s *t*‐test. *P < 0.01. Levels of α‐tubulin were monitored as a loading control for whole‐cell extracts. Among several variants of Ets1, p54 isoform was used for these experiments (B–D) (see Fig. 3A).

We have previously reported that the induction of Snail by TGF‐β is synergistically promoted by active Ras mutants such as H‐RasG12V and K‐RasG12D and that cancer cells harboring an active Ras mutation show dramatic induction of Snail following TGF‐β stimulation alone [15, 22]. The expression of ZEB1 is reportedly regulated by Ets1 in breast cancer cells and positively correlated with the mesenchymal phenotypes in breast cancer and OSCC cells [8, 13]. In this study, we determined the role of Ets1 in Snail induction by TGF‐β in cooperation with RasG12V. Similar to our previous report [15], Snail promoter activity was synergistically enhanced by TGF‐β in HeLa cells expressing RasG12V (Fig. 1A). Overexpression of Ets1 further promoted Snail promoter activity through TGF‐β in combination with RasG12V (Fig. 1B). Because the transfection efficiency of HeLa cells was almost 80% as determined by immunostaining [15], expression of endogenous Snail protein was verified by immunoblotting analysis upon transfection with RasG12V in response to TGF‐β. Like the Snail promoter activity, Snail was upregulated by TGF‐β in combination with RasG12V [15], which was further enhanced by ectopic expression of Ets1 (Fig. 1C). In addition to RasG12V, HGF also promoted Snail induction by TGF‐β [15], which was further enhanced by Ets1 (Fig. S1A). Ets2, a paralog of the *Ets1* gene, also activated the Snail promoter to a greater extent than Ets1 (Fig. 1D), and enhanced expression of endogenous Snail protein induced by TGF‐β in combination with RasG12V in HeLa cells (Fig. 1E). Thus, these findings suggest that Ets1/2 is involved in Snail induction in response to TGF‐β and RasG12V.

### siRNAs against both Ets1 and Ets2, but not either alone, inhibit Snail induction by TGF‐β

**Fig. 2:**
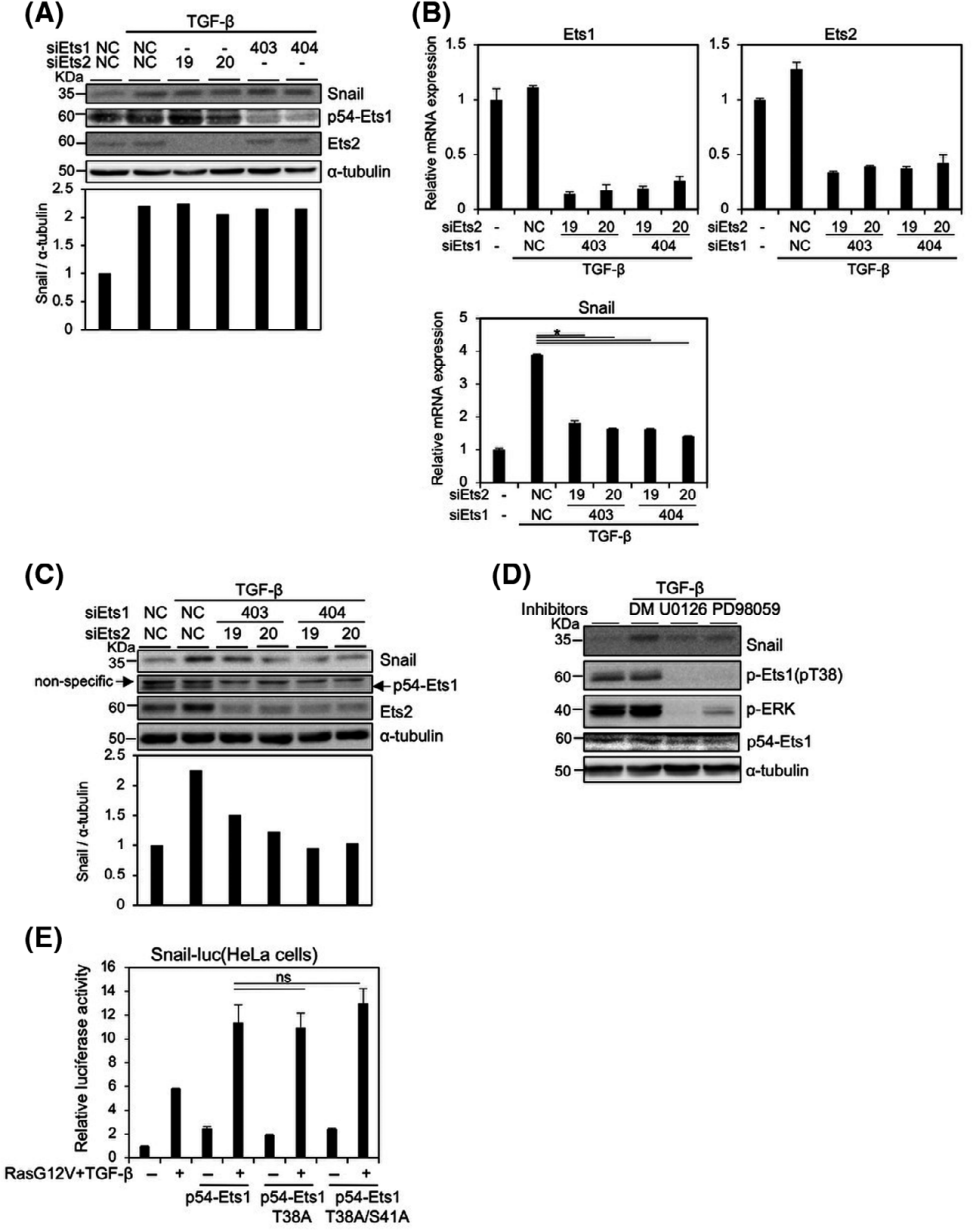
Involvement of Ets1/2 in Snail induction by TGF‐β in Panc‐1 cells. (A–C) Panc‐1 cells transfected with two different siRNAs against Ets1 (403 and 404) and Ets2 (19 and 20) or control siRNA (NC) were treated with 1 ng·mL^−1^ TGF‐β1 for 3 h (A and C) or 1 h (B), followed by immunoblot analysis using the indicated antibodies (A and C) and qPCR analysis (B). (D) Panc‐1 cells were pretreated with 10 μm of the indicated MEK inhibitors for 24 h and treated with 1 ng·mL^−1^ TGF‐β1 for 3 h, followed by immunoblot analysis using the indicated antibodies. Levels of α‐tubulin were monitored as a loading control for whole‐cell extracts (A, C, and D), and the ratio of Snail to α‐tubulin was validated by densitometric analysis and shown at the bottom (A and C). (E) After transfection with the indicated expression plasmids in HeLa cells, Snail‐Luc activities were measured. Each value represents the mean ± SD of three biological replicates. Similar results were obtained in at least three independent experiments. *P* values were determined by Student’s *t*‐test. *P < 0.01. ns, not significant.

Panc‐1 cells have the K‐RasG12D mutation, which results in constitutive activation of Ras signaling. Short‐term treatment (3 h) with TGF‐β also induced the expression of Snail protein and activated Smad2/3 phosphorylation on the C‐terminal SSXS motif in Panc‐1 cells (Fig. S1B) [15]. After transfection with siRNAs against either Ets1 (siEts1) or Ets2 (siEts2) alone, Snail induction by TGF‐β was not dramatically suppressed (Fig. 2A). However, transfection with both siEts1 and siEts2 inhibited Snail induction by TGF‐β at the mRNA and protein levels (Fig. 2B,C). Treatment with MEK inhibitors, U0126 and PD98059, considerably inhibited Snail induction by TGF‐β, which was accompanied by reduced levels of phospho‐ERK, phospho‐Ets1, as well as Ets1 expression. It has been reported that ERK1/2 phosphorylates Ets1 at threonine 38 (T38) and serine 41 (S41) [11]. After these residues were substituted with alanine residues, Ets1‐T38A and Ets1‐T38A/S41A still enhanced Snail promoter activity by combined treatment with TGF‐β and RasG12V (Fig. 2E). Indeed, TGF‐β treatment did not affect the phosphorylation status of Ets1 and ERK (Fig. 2D), suggesting that the phosphorylation of Ets1 at T38 and S41 is dispensable for Snail induction by TGF‐β.

### Snail induction by Ets1 variants

**Fig. 3:**
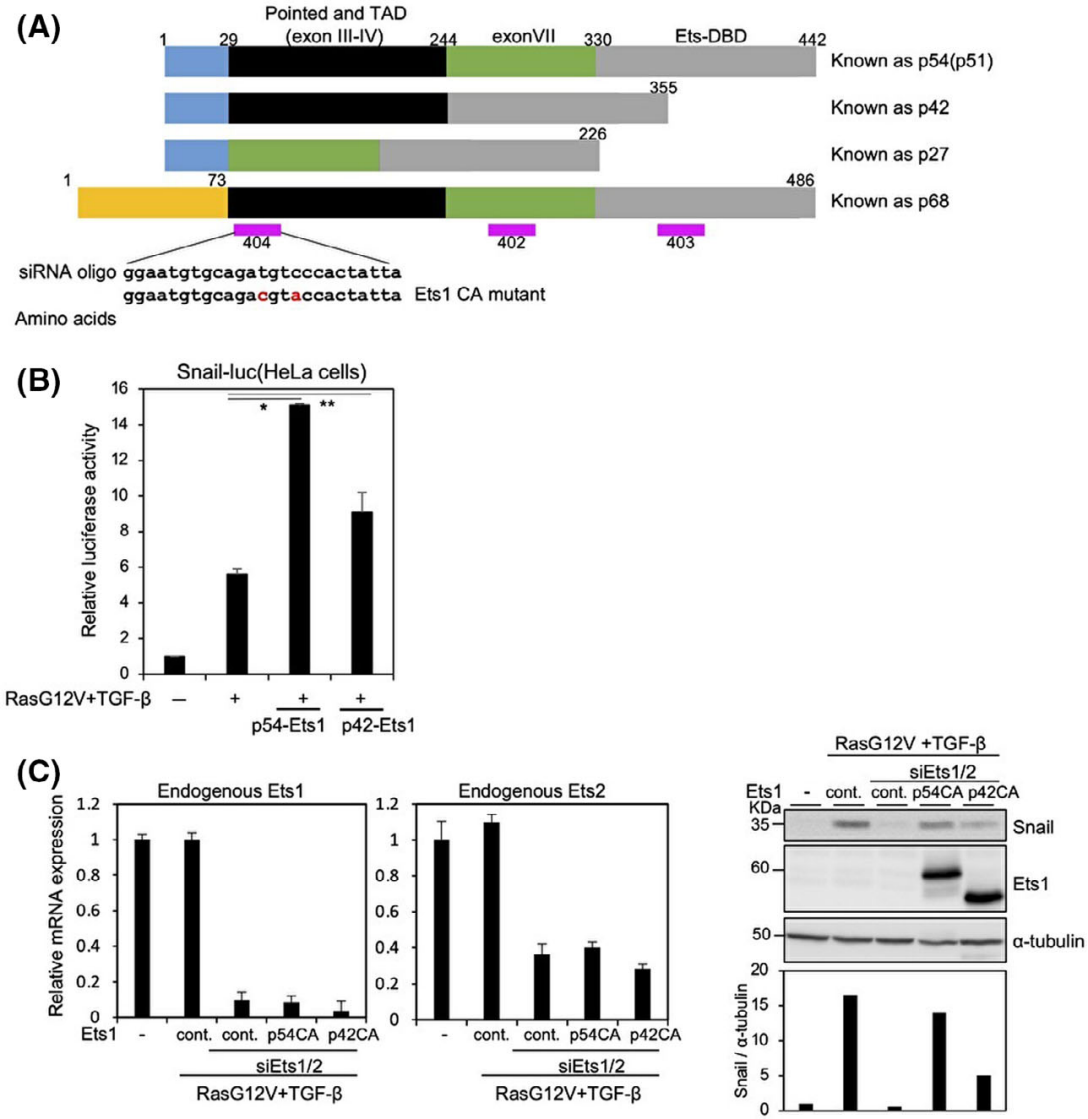
p54‐Ets1, rather than p42‐Ets1, enhances Snail induction by RasG12V and TGF‐β. (A) Schematic illustration of an alternative promoter (p68) and splicing alternative variants (p54, p42, and p27) of Ets1 and three kinds of siRNAs against Ets1 (pink). Point mutations in the siRNA (404) target sequence are shown in red. Pointed—the pointed domain; TAD—the transactivation domain; Ets‐DBD—the Ets‐DNA binding domain. (B) HeLa cells were co‐transfected with mouse Snail promoter–reporter construct (Snail‐Luc.) and the indicated expression plasmids. At 8 h after transfection, the cells were treated with 1 ng·mL^−1^ TGF‐β1 for an additional 18 h, and the activities of Snail promoters were measured. (C) siRNAs against both Ets1 (404) and Ets2 (20) were transfected in HeLa cells in combination with the indicated plasmids. After treatment with 1 ng·mL^−1^ TGF‐β for 3 h, qPCR analysis (left two panels) and immunoblot analysis using the indicated antibodies (right) was performed. To amplify endogenous Ets1, primers for qPCR were prepared in Ets‐DBD and 3’non‐coding regions. Levels of α‐tubulin were monitored as a loading control for whole‐cell extracts, and the ratio of Snail to α‐tubulin was validated by densitometric analysis and shown at the bottom (C). p54CA—siRNA (404)‐resistant p54‐Ets1; p42CA—siRNA (404)‐resistant p42‐Ets1; cont.—control plasmid. Each value represents the mean ± SD of three biological replicates. Similar results were obtained in at least three independent experiments. *P* values were determined by Student’s *t*‐test. *P < 0.01, **P < 0.1.

The p68 isoform of Ets1 is derived from an alternate promoter, whereas alternative splicing in Ets1 produces p54(p51)‐Ets1 including the exon VII domain, p42‐Ets1 excluding the exon VII domain, and p27‐Ets1 lacking the sequences encoded by exons III–VI (Fig. 3A) [11]. The inclusion of the DNA binding domain, but not the transactivation domain, allows p27‐Ets1 to act as a transdominant‐negative regulator of Ets1‐dependent transcription [11]. In OSCC cells, p68‐Ets1 is ubiquitously expressed, and the levels of p54‐Ets1 were much higher than those of p42‐Ets1 as determined by conventional PCR analysis (Fig. S1C,D). The Snail promoter luciferase assay revealed that p54‐Ets1 promoted Snail promoter activity by RasG12V and TGF‐β, whereas p42‐Ets1 had less of an effect (Fig. 3B). It is known that transcriptional activation of Ets1 can lead to an increase in its own expression [23]. After we evaluated the specificity of three kinds of siRNAs in some cells (Fig. S1E–G), we generated an Ets1‐CA mutant in which the siRNA (404) target sequence was mutated without affecting the amino acid sequence (Fig. 3A). Ets1‐CA mutants in p54‐ and p42‐Ets1 exhibited resistance to the siRNA (404) against Ets1 (Fig. S1H). Upon transfection with both siEts1 (404) and siEts2, p54CA‐Ets1 markedly upregulated the levels of endogenous Snail protein compared with p42CA‐Ets1 (Fig. 3C). p27‐Ets1 also negatively dominated the Snail promoter activity (Fig. S1I), whereas p68‐Ets1 was not precisely evaluated due to the unstable expression of its protein after transient transfection. These findings suggest that p54‐Ets1, rather than p42‐Ets1, is involved in Snail expression.

### Snail downregulation by siEts1/2 in OSCC cells

**Fig. 4:**
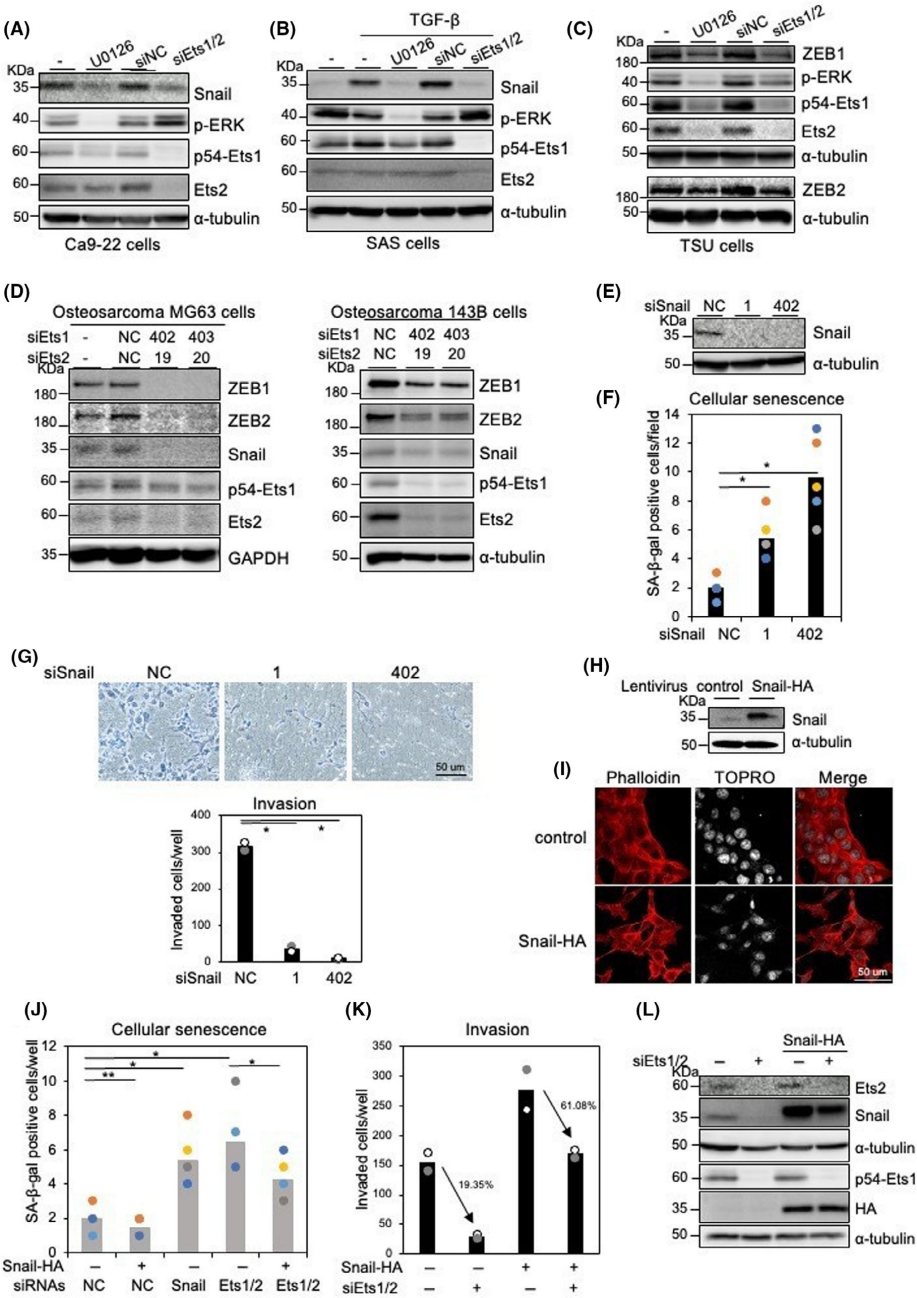
Involvement of Ets1/2 in Snail expression in Ca9‐22 cells. (A–C) The cells were treated with 10 μm MEK inhibitor (U0126) for 24 h or transfected with siRNAs against both Ets1 (404) and Ets2 (19) or control siRNA (siNC), followed by immunoblot analysis using the indicated antibodies. SAS cells were treated with 1 ng·mL^−1^ TGF‐β1 for 3 h (B). (D) siRNAs against Ets1 (402 and 403) and Ets2 (19 and 20) or control siRNA were transfected in osteosarcoma MG63 and 143B cells, followed by immunoblot analysis using the indicated antibodies. (E–G) Ca9‐22 cells transfected with Snail siRNAs (1 and 402) or control siRNA (NC) were subjected to immunoblot analysis (E), cellular senescence assay (F), and invasion assay (G). (H, I) Ca9‐22 cells infected with lentiviruses carrying control or HA‐tagged Snail were subjected to immunoblot analysis (H) and immunofluorescence analysis using the indicated antibodies (I). (J–L) siRNAs against both Ets1 (404) and Ets2 (19) were transfected in Snail‐overexpressed Ca9‐22 cells, followed by cellular senescence assay (J), invasion assay (K), and immunoblot analysis (L). α‐tubulin levels were monitored as a loading control (A, B, C, D, E, and L). Each value represents the mean ± SD of three biological replicates. Similar results were obtained in at least three independent experiments. *P* values were determined by Student’s *t*‐test. *P < 0.01, **P < 0.1. Scale bars = 50 μm.

In OSCC cells, Snail is highly expressed in OSCC Ca9‐22 cells [24]. When Ca9‐22 cells were treated with a MEK1/2 inhibitor, U0126, or transfected with siEts1/2, Snail was considerably downregulated (Fig. 4A). Intriguingly, SB431542, a TGF‐β type I receptor inhibitor, also reduced the expression of Snail in the cell (Fig. S2A). In HeLa cells, Snail was induced by both TGF‐β and active Ras signals, whereas OSCC SAS and Ca9‐22 cells exhibited Snail induction by TGF‐β alone, likely due to constitutively activated ERK signals without active *K‐Ras* mutations (Fig. S2B). Snail induction by TGF‐β in SAS cells was also repressed by U0126 and siEts1/2 (Fig. 4B). Additionally, siEts1/2 reduced steady levels of ZEB1/2 in OSCC TSU cells (Fig. 4C). We next examined whether siEts1/2 suppressed both Snail and ZEB1/2, both of which were constitutively expressed in cancer cells. In almost all OSCC cells we tested, either Snail or ZEB1/2, but not both, was expressed [8]. By contrast, osteosarcoma MG63 and 143B cells, and breast cancer HCC 1395 cells, constitutively expressed both Snail and ZEB1/2 [7]. When Ets1/2 were knocked down in these cells, expression of Snail, as well as ZEB1/2, were suppressed (Fig. 4D and Fig. S2C), suggesting that Ets1/2 maintains expression of not only Snail but also ZEB1/2 in cancer cells.

ZEB1/2 are positively correlated with EMT phenotypes, whereas Snail is not positively correlated with the mesenchymal phenotypes of breast cancer and OSCC cells [7, 8]. We previously reported that the silencing of Snail confers cellular senescence in normal fibroblasts [21]. Cellular senescence of Ca9‐22 cells was enhanced by Snail siRNAs without affecting mRNA expressions of CDK inhibitors such as p16INK4A and p21CIP1 (Fig. 4E,F, and Fig. S2D). Similar to previous reports in OSCC cells [25], motile properties were also suppressed by siRNAs against Snail (Fig. 4G). By contrast, Ca9‐22 cells overexpressing Snail exhibited stress fiber formation (Fig. 4H,I), suppression of cellular senescence, and enhancement of invasion (Fig. 4J,K). When Ets1/2 was knocked down in Ca9‐22 cells overexpressing Snail, a number of senescent and invaded cells were partially rescued (Fig. 4J–L), suggesting that Ets1/2 regulates invasion and cellular senescence properties through, at least in part, Snail in OSCC cells.

## Discussion

In the present study, we found that Ets1/2 regulates the expression of representative key regulators for EMT, Snail, and ZEB1/2. In breast cancer and OSCC cells, expression profiles of ZEB1/2 and Snail are not positively correlated with each other and those of Ets1/2 levels (Fig. S3A) [8]. In the case of ZEB1/2, Ets homologous factor (EHF), the epithelium‐specific subfamily of the Ets family, is a negative regulator for Ets1/2 [8] and is markedly negatively correlated with the expression of ZEB1/2 [8, 13]. However, the expression of EHF is not correlated with that of Snail in OSCC and breast cancer cells. Except for sarcoma and HCC 1395 cells (Fig. 4D and Fig. S2C), carcinoma cells with upregulation of both Snail and ZEB1/2 are not so frequently observed [7, 8]. Thus, both key regulators of EMT could not be overexpressed simultaneously through unknown mechanisms, likely via regulating the expression of microRNAs.

Expression of ZEB1/2 is reciprocal to the epithelial markers, such as E‐cadherin and epithelial splicing regulatory protein (ESRP), and positively correlated with the mesenchymal markers such as N‐cadherin and vimentin, whereas the expression of Snail is not correlated with these EMT markers. Numerous papers have reported that gene silencing of ZEB1, as well as Snail, inhibits migration and invasion of various kinds of cancer cells [3, 26]. However, the function of Snail and ZEB1/2 during cancer progression is still controversial. In mouse cancer models, genetic depletion of ZEB1 in the pancreas is shown to reduce undifferentiated carcinomas, invasion, and metastasis [27]. By contrast, Zheng et al. reported in the same mouse pancreas cancer model that Snail depletion does not affect tumor differentiation, invasion, and metastasis, but contributes to enhanced sensitivity to chemotherapy [28]. In breast cancer, inhibiting EMT by overexpressing miR‐200, a well‐known microRNA that directly targets ZEB1/2, does not affect lung metastasis, but contributes to recurrent lung metastasis after chemotherapy [29]. Thus, it seems that EMT‐TFs have specific functions, which are not redundant but are instead complementary. Moreover, functions of EMT‐TFs can be tissue specific, as demonstrated by the different roles of Snail in metastasis of various kinds of cancer [30]. Importantly, we found that Snail siRNAs promote cellular senescence much more than ZEB1/2 siRNAs in normal fibroblast IMR90 cells without upregulation of p16INK4A (Fig. S3B–D). It is reported that Ets1/2 induces cellular senescence by activating the p16INK4A promoter in human diploid fibroblasts [31]. These findings suggest that cellular senescence by Ets1/2 is defined by the molecular balance between cyclin inhibitors and Snail. The pathophysiological significance of cellular senescence in cancer cells is not well understood, though Snail could act as an EMT‐TF to promote tumor aggressiveness through, at least in part, regulating cellular senescence.

Ets1/2 promoted Snail promoter activation (Fig. 1). Several typical Ets‐binding sites and putative Ets‐binding sites are contained in the Snail promoter luciferase construct. However, a mutation in these Ets‐binding sites did not affect the Snail promoter activity by ectopic Ets1 (Fig. S3E). In mouse embryonic fibroblasts, deletion of both Ets1 and Ets2, but not either alone, is necessary to inhibit transformation by Ha‐RasG12V [32]. Ha‐RasG12V induces a robust increase in c‐Myc expression by promoting the binding of Ets1/2 to the GGGAAA site in the c‐Myc promoter. Since the ZEB1 promoter luciferase construct harboring mutations in the two putative binding sites was not activated by Ets1 [13], Snail expression appears to be regulated by unknown binding sites of Ets1 in the Snail promoter region.

In our previous study, short‐term treatment with U0126 failed to inhibit Snail mRNA induction by TGF‐β in cooperation with active Ras [15], whereas long‐term treatment could do so when accompanied by Ets1 downregulation (Figs 2D and 4A–C). Indeed, Snail induction by ectopic expression of Ets1 was reduced by MEK‐ERK inhibitors and Ets1 phosphorylation site mutants (T38A and T38A/S41A) could enhance Snail promoter activity (Fig. 2E), suggesting that Snail expression appears to be regulated by post‐translational modifications of Ets1 other than T38 and S41 phosphorylation, which are highly dependent on the MEK‐ERK pathway.

We also found that p54‐Ets1, but not p42‐Ets1, enhances Snail expression, suggesting that exon VII in Ets1 has a crucial role in Snail expression. Like the Snail promoter, the Stromelysin‐1 gene promoter is activated by p54‐Ets1, but marginally by p42‐Ets1 [33]. The exon VII domain is reported to specifically interact with the POU‐domain transcription factor Pit‐1 to synergistically activate the prolactin promoter [34, 35]. Hypoxia‐inducible factor‐2 α (HIF‐2α) cooperates with Ets1 in activating transcription of the vascular endothelial growth factor receptor‐2 by interacting with the exon VII domain [36]. In addition, Runt‐related transcription factor 1 (RUNX1) synergistically activates platelet factor 4 expression along with Ets family proteins through the exon VII domain [37, 38]. These exon VII‐binding proteins, including unidentified proteins, could also be involved in Snail induction and EMT‐associated cancer progression. The underlying molecular mechanisms will need to be elucidated in the near future.

In conclusion, among key EMT transcription factors, ZEB1/2 are positively correlated with the mesenchymal marker proteins, whereas Snail is not correlated with EMT marker proteins, but largely regulates cellular senescence (Fig. S3F). Importantly, Ets1/2 regulates both representative EMT regulators of Snail and ZEB1/2 in cancer cells.

## Conflict of interest

The authors declare no conflict of interest.

## Author contributions

MKI, KE, YI, AHO, and YK performed experiments and analyzed data. KU, KY, and KM drafted the manuscript. MS conceived and designed the project and drafted the manuscript.

## Supporting information


**Fig. S1.** Expression profiles of Ets1 variants in OSCC cells. A: Endogenous levels of Snail in HeLa cells overexpressing Ets1 were determined by immunoblotting at 3 h after combined treatment with 1 ng/mL of TGF‐β1 and 10 ng/mL of HGF. B: Phosphorylation of Smad2/3 was determined by immunoblotting at 3 and 24 h after treatment with 1 ng/mL of TGF‐β1 in Panc‐1 cells. C: Schematic illustration of the alternative promoter (p68) and splicing alternative variants (p54, p42, and p27) of Ets1 and primers for conventional PCR (black arrows) and qPCR (blue arrows). D: mRNA levels of Ets1 variants in OSCC cells were analyzed by conventional PCR. The ratio of p54, p42, p27 or p68 to GAPDH was validated by densitometric analysis and shown at the bottom. The value of the SAS cells is indicated as “1”. E, F, and G: After transfection with three kinds of siRNAs against Ets1 (402, 403, and 404) in breast cancer (MDA‐MB231) and OSCC (HSC2 and TSU) cells, the suppressive effects of the siRNAs were evaluated by conventional PCR (E), qPCR (F), and immunoblot analyses (G). The ratio of p54, p42 or p27 to GAPDH was validated by densitometric analysis and shown at the bottom (E). H: HeLa cells transfected with the indicated expression plasmids were re‐transfected with siRNA against Ets1 (404) and subjected to immunoblot analysis. Levels of α‐tubulin were monitored as a loading control for whole‐cell extracts. I: HeLa cells were cotransfected with the indicated expression plasmids. At 8 h after transfection, the cells were treated with TGF‐β1 for an additional 18 h, and the activities of Snail promoters were measured. Each value represents the mean ± s.d. of three biological replicates. Similar results were obtained in at least three independent experiments.
**Fig. S2.** Downregulation of both Snail and ZEB1/2 by siEts1/2. A: Ca9‐22 cells were treated with the indicated concentration of SB431542, a TGF‐β type I receptor inhibitor, for 48 h, and subjected to immunoblotting analysis. B: HeLa, Ca9‐22, and SAS cells were treated with 1 ng/mL TGF‐β1 for 24 h, followed by immunoblot analysis using the indicated antibodies. C: Breast cancer HCC1395 cells were transfected with siRNAs against both Ets1 (402 and 403) and Ets2 (19 and 20) or control siRNA (NC), and then subjected to immunoblot analysis using the indicated antibodies. D: mRNA levels of p16INK4A and p21CIP1 were analyzed by qPCR, after transfection with siRNAs against both Ets1 (404) and Ets2 (19) or control siRNA (NC). Each value represents the mean ± s.d. of three biological replicates. Similar results were obtained in at least three independent experiments. *p* values were determined by Student’s *t*‐test. ns, not significant.
**Fig. S3.** Cellular senescence in normal fibroblast IMR90 cells. A: In cancerous tissues from patients with breast cancer in the TCGA dataset (n = 1299), *Snail* mRNA levels were compared with *ZEB1/2* mRNA levels. B, C, and D: Normal fibroblast IMR90 cells transfected with control siRNA (siNC), siSnail, or siZEB1/2 were subjected to immunoblot analysis (B), qPCR analysis (C), and cellular senescence assay (D). α‐tubulin levels were monitored as a loading control (B). E: Point mutations in the Ets1‐binding sites (EBS) in the human Snail promoter reporter construct are shown in red. After transfection with the indicated plasmids, luciferase activities were measured. F: Schematic illustration of Ets’s role in regulation of Snail and ZEB1/2 during EMT. Each value represents the mean ± s.d. of three biological replicates. Similar results were obtained in at least three independent experiments. *p* values were determined by Student’s *t*‐test. *p < 0.01. Scale bars = 50 μm.
**Table S1.** PCR primer pairs used in this study.

## Data Availability

The data in the present study are available from the corresponding author on reasonable request.
