# Supplementary material for: Ets family proteins regulate the EMT transcription factors Snail and ZEB in cancer cells
Source: FEBS Open Bio. 2022 Apr 29;12(7):1353–64. doi: 10.1002/2211-5463.13415 (PMC9249322; doi:10.1002/2211-5463.13415)
Supplement: Supplementary file 1 — Fig. S1. Expression profiles of Ets1 variants in OSCC cells. A: Endogenous levels of Snail in HeLa cells overexpressing Ets1 were determined by immunoblotting at 3 h after combined treatment with 1 ng/mL of TGF‐β1 and 10 ng/mL of HGF. B: Phosphorylation of Smad2/3 was determined by immunoblotting at 3 and 24 h after treatment with 1 ng/mL of TGF‐β1 in Panc‐1 cells. C: Schematic illustration of the alternative promoter (p68) and splicing alternative variants (p54, p42, and p27) of Ets1 and primers for conventional PCR (black arrows) and qPCR (blue arrows). D: mRNA levels of Ets1 variants in OSCC cells were analyzed by conventional PCR. The ratio of p54, p42, p27 or p68 to GAPDH was validated by densitometric analysis and shown at the bottom. The value of the SAS cells is indicated as “1”. E, F, and G: After transfection with three kinds of siRNAs against Ets1 (402, 403, and 404) in breast cancer (MDA‐MB231) and OSCC (HSC2 and TSU) cells, the suppressive effects of the siRNAs were evaluated by conventional PCR (E), qPCR (F), and immunoblot analyses (G). The ratio of p54, p42 or p27 to GAPDH was validated by densitometric analysis and shown at the bottom (E). H: HeLa cells transfected with the indicated expression plasmids were re‐transfected with siRNA against Ets1 (404) and subjected to immunoblot analysis. Levels of α‐tubulin were monitored as a loading control for whole‐cell extracts. I: HeLa cells were cotransfected with the indicated expression plasmids. At 8 h after transfection, the cells were treated with TGF‐β1 for an additional 18 h, and the activities of Snail promoters were measured. Each value represents the mean ± s.d. of three biological replicates. Similar results were obtained in at least three independent experiments. Fig. S2. Downregulation of both Snail and ZEB1/2 by siEts1/2. A: Ca9‐22 cells were treated with the indicated concentration of SB431542, a TGF‐β type I receptor inhibitor, for 48 h, and subjected to immunoblotting analysis. B: HeLa, Ca9 [file FEB4-12-1353-s001.pdf]

**Supplementary Information**

**Figure Legends**

**Supplementary Figure S1. Expression profiles of Ets1 variants in OSCC cells.** A: Endogenous levels of Snail in HeLa cells overexpressing Ets1 were determined by immunoblotting at 3 h after combined treatment with 1 ng/mL of TGF-β1 and 10 ng/mL of HGF. B: Phosphorylation of Smad2/3 was determined by immunoblotting at 3 and 24 h after treatment with 1 ng/mL of TGF-β1 in Panc-1 cells. C: Schematic illustration of the alternative promoter (p68) and splicing alternative variants (p54, p42, and p27) of Ets1 and primers for conventional PCR (black arrows) and qPCR (blue arrows). D: mRNA levels of Ets1 variants in OSCC cells were analyzed by conventional PCR. The ratio of p54, p42, p27 or p68 to GAPDH was validated by densitometric analysis and shown at the bottom. The value of the SAS cells is indicated as "1". E, F, and G: After transfection with three kinds of siRNAs against Ets1 (402, 403, and 404) in breast cancer (MDA-MB231) and OSCC (HSC2 and TSU) cells, the suppressive effects of the siRNAs were evaluated by conventional PCR (E), qPCR (F), and immunoblot analyses (G). The ratio of p54, p42 or p27 to GAPDH was validated by densitometric analysis and shown at the bottom (E). H: HeLa cells transfected with the indicated expression plasmids were re-transfected with siRNA against Ets1 (404) and subjected to immunoblot analysis. Levels of α-tubulin were monitored as a loading control for whole-cell extracts. I: HeLa cells were cotransfected with the indicated expression plasmids. At 8 h after transfection, the cells were treated with TGF-β1 for an additional 18 h, and the activities of Snail promoters were measured. Each value represents the mean ± s.d. of three biological replicates. Similar results were obtained in at least three independent experiments.

**Supplementary Figure S2. Downregulation of both Snail and ZEB1/2 by siEts1/2.** A: Ca9-22 cells were treated with the indicated concentration of SB431542, a TGF-β type I receptor inhibitor, for 48 h, and subjected to immunoblotting analysis. B: HeLa, Ca9-22, and SAS cells were treated with 1 ng/mL TGF-β1 for 24 h, followed by immunoblot analysis using the indicated antibodies. C: Breast cancer HCC1395 cells were transfected with siRNAs against both Ets1 (402 and 403) and Ets2 (19 and 20) or control siRNA (NC), and then subjected to immunoblot analysis using the indicated antibodies. D: mRNA levels of p16INK4A and p21CIP1 were analyzed by qPCR, after transfection with siRNAs against both Ets1 (404) and Ets2 (19) or control siRNA

(NC). Each value represents the mean ± s.d. of three biological replicates. Similar results were obtained in at least three independent experiments. *p* values were determined by Student's t-test. ns, not significant.

**Supplementary Figure S3. Cellular senescence in normal fibroblast IMR90 cells.** A: In cancerous tissues from patients with breast cancer in the TCGA dataset (n=1299), *Snail* mRNA levels were compared with *ZEB1/2* mRNA levels. B, C, and D: Normal fibroblast IMR90 cells transfected with control siRNA (siNC), siSnail, or siZEB1/2 were subjected to immunoblot analysis (B), qPCR analysis (C), and cellular senescence assay (D). α-tubulin levels were monitored as a loading control (B). E: Point mutations in the Ets1-binding sites (EBS) in the human Snail promoter reporter construct are shown in red. After transfection with the indicated plasmids, luciferase activities were measured. F: Schematic illustration of Ets's role in regulation of Snail and ZEB1/2 during EMT. Each value represents the mean ± s.d. of three biological replicates. Similar results were obtained in at least three independent experiments. *p* values were determined by Student's t-test. *$p < 0.01$. Scale bars = 50 μm.

**Supplementary Table S1**

PCR primer pairs used in this study.

# Supplementary Figure S1

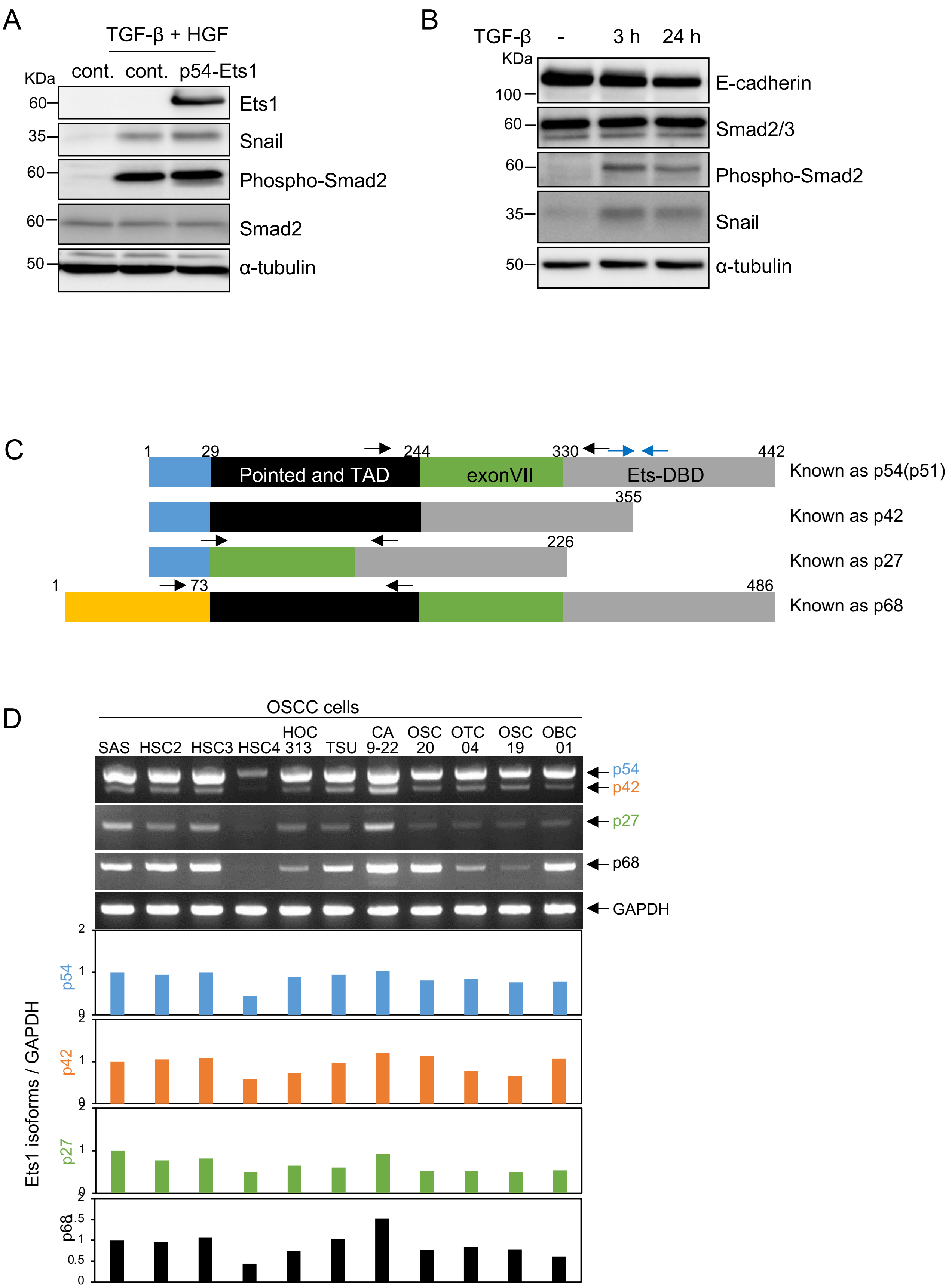

# Supplementary Figure S1

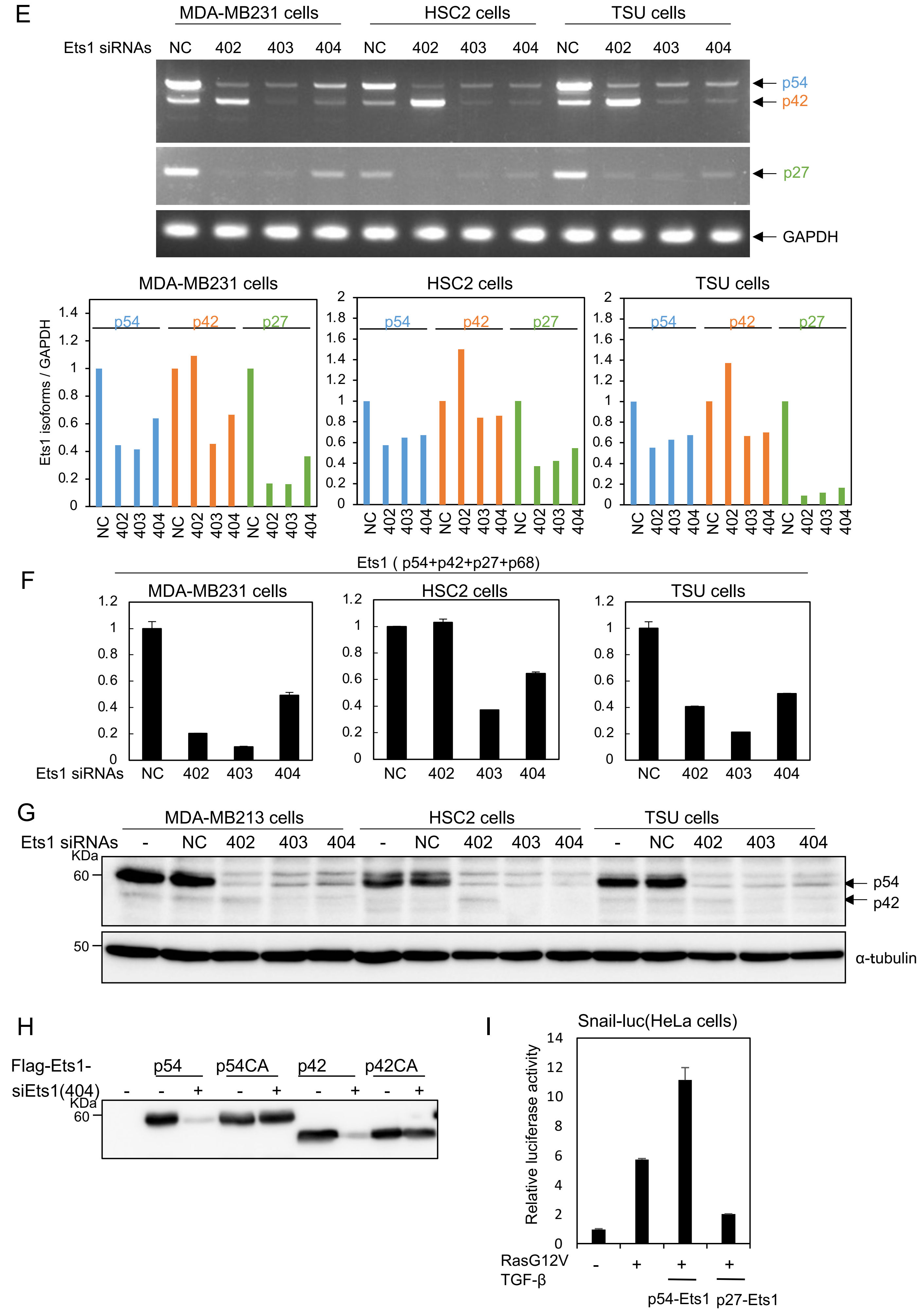

Supplementary Figure S2

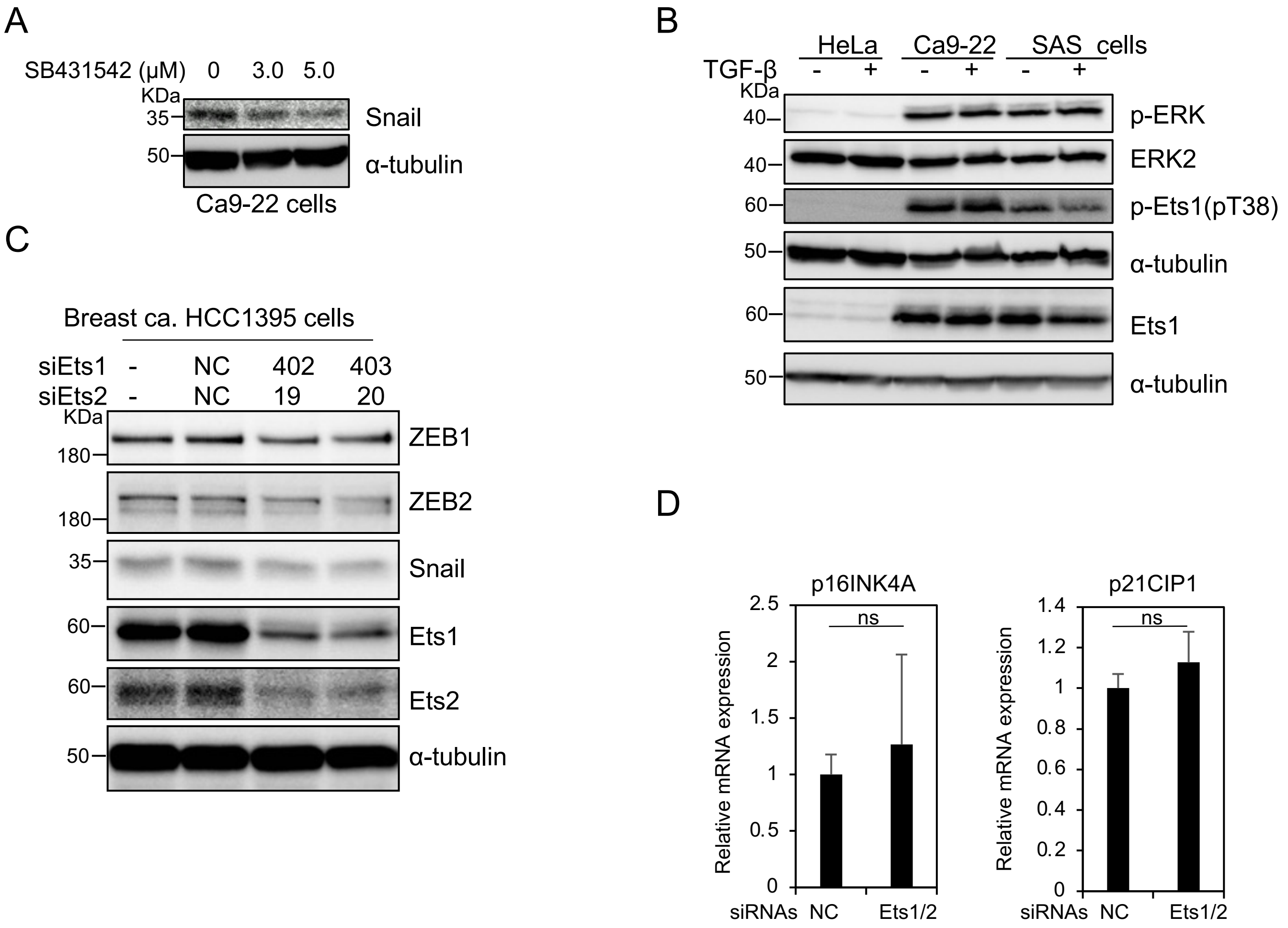

# Supplementary Figure S3

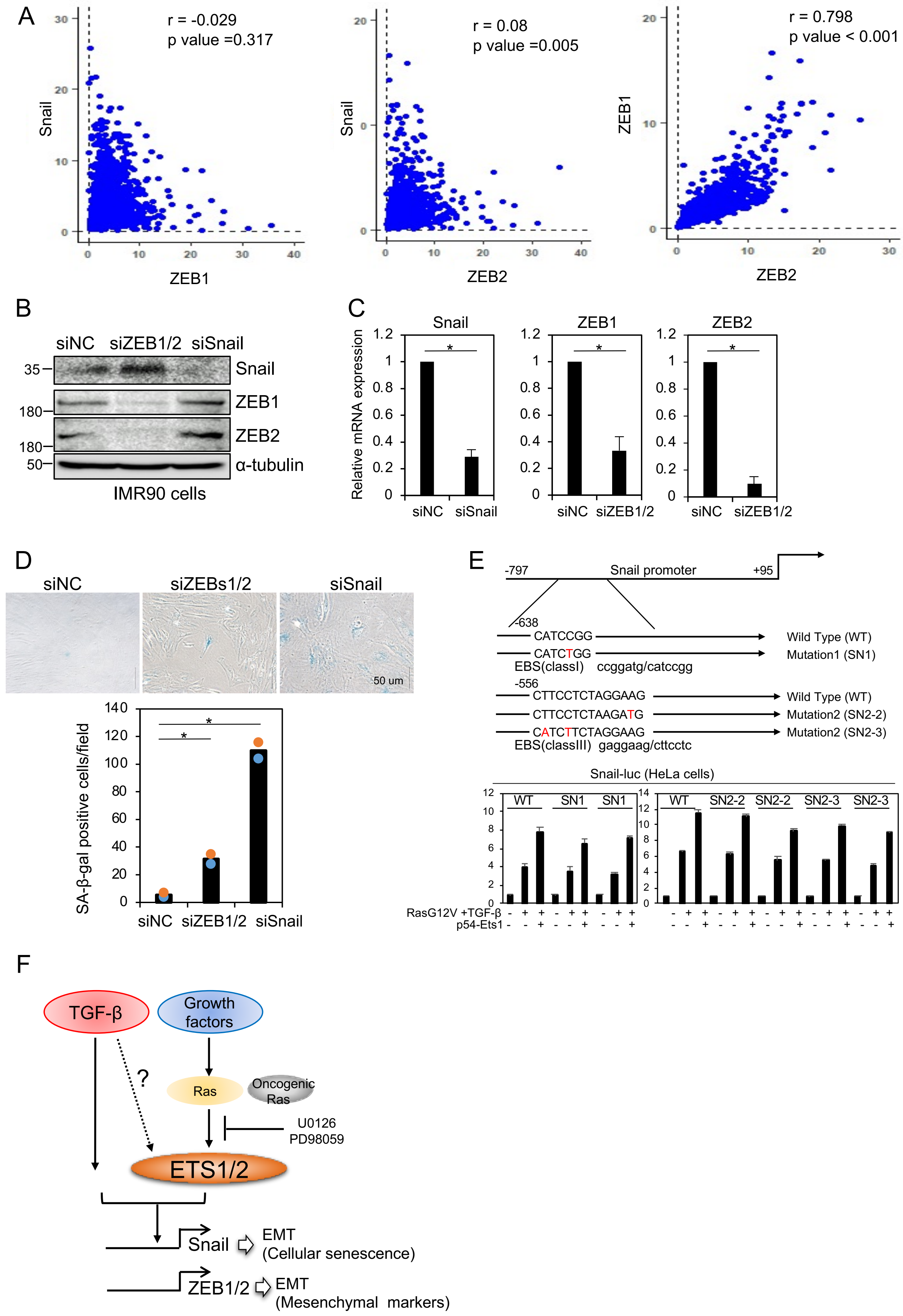

# Supplementary Table S1

| Conventional PCR | | Forward (5' to 3') | Reverse (5' to 3') |
|---|---|---|---|
| GAPDH | | CGACCACTTTGTCAAGCTCA | CCCTGTTGCTGTAGCCAAAT |
| p68 | | GAACTGTGGTTTCCAGTCCAA | TGGGACATCTGCACATTCCA |
| p54 | | TGACTACCCCTCGGTCATTC | AACTGCCATAGCTGGATTGG |
| p42 | | TGACTACCCCTCGGTCATTC | AACTGCCATAGCTGGATTGG |
| p27 | | CCTCCCCCGGTAAGCTCGG | AACTGCCATAGCTGGATTGG |
| | | | |
| **Quantitative real-time PCR (qPCR)** | | | |
| Snail | | TTCTCACTGCCATGGAATTCC | GCAGAGGACACAGAACCAGAAA |
| Ets1 | | CCCGTACGTCCCCCACTCCT | TGGGACATCTGCACATTCCA |
| Ets2 | | TTTCTCATGACTCCGCCAACT | GGCTTGACTCATCACAGCCTT |
| GAPDH | | CGACCACTTTGTCAAGCTCA | CCCTGTTGCTGTAGCCAAAT |
| ZEB1 | | CAATGATCAGCCTCAATCTGCA | CCATTGGTGGTTGATCCCA |
| ZEB2 | | AAGCCCCATCAACCCATACAAG | AAATTCCTGAGGAAGGCCCA |
| p16 | | GCACCAGAGGCAGTAACCAT | CTTTCAATCGGGGATGTCTG |
| p21 | | GGAAGACCATGTGGACCTGT | GGCGTTTGGAGTGGTAGAAA |
| | | | |
| **DNA constructs and mutagenesis** | | | |
| Ets1-CA mutation | | ATGGAATGTGCAGACGTACCACTATTAACTCCA | TGGAGTTAATAGTGGTACGTCTGCACATTCCAT |
| Ets1-T38A/S41A | | TATTAGCTCCAAGCGCCAAAGAAATGATGT | ACATCATTTCTTTGGCGCTTGGAGCTAATA |
